# HIV as a chronic disease considerations for service planning in resource-poor settings

**DOI:** 10.1186/1744-8603-7-35

**Published:** 2011-10-04

**Authors:** Lucy Reynolds

**Affiliations:** 1 Faculty of Public Health Policy, London School of Hygiene and Tropical Medicine, London, WC1H 9SH, UK

**Keywords:** HIV, Access to essential medicines, Adherence, Antiretroviral, Fees, Stigma, Infection control, Chronic disease, Intellectual property, Criminalisation

## Abstract

This paper reviews the healthcare issues facing nations which have a substantial caseload of chronic HIV cases. It considers the challenges of extending antiretroviral coverage to an expanding caseload as supplier price rises and international trade agreements come into force to reduce the availability of affordable antiretrovirals just as the economic downturn restricts donor funding. It goes on to review the importance in this context of supporting adherence to drug regimens in order to preserve access to affordable antiretrovirals for those already on treatment, and of removing key barriers such as patient fees and supply interruptions. The demands of those with chronic HIV for health services other than antiretroviral therapy are considered in the light of the fearful or discriminatory attitudes of non-specialist healthcare staff due to HIV-related stigma, which is linked with the weakness of infection control measures in many health facilities. The implications for prevention strategies including those involving criminalisation of HIV transmission or exposure are briefly summarised for the current context, in which the caseload of those whose chronic HIV infection must be controlled with antiretrovirals will continue to rise for the foreseeable future.

## Introduction

In 2009, an estimated 33.3 million [31.4 million-35.3 million] people were living with HIV, according to UNAIDS[1]. With successful antiretroviral treatment, life expectancy for people living with HIV (PLHIV) can be restored to near normal: thus HIV has latterly been transformed into a manageable chronic illness, compatible with fairly good health, lifestyle and economic participation. Most countries now have from a few to many thousands of their population maintained with chronic HIV infection on antiretroviral treatment (ART). This situation already causes some significant challenges, which will increase as the ongoing spread of HIV adds to the caseload. Much has been written about the need to introduce and scale-up antiretroviral treatment to prevent deaths from AIDS. Much less has been said about planning for the situation when PLHIV have been stabilised on treatment so that their immunity is largely restored and they can resume familial and social roles, although a number of important medical and social issues emerge at this stage. This paper aims to raise awareness of some of the key questions for health ministries and governments.

## Discussion

### Scope of coverage

As HIV prevalence continues to rise through the roll-out of highly active antiretroviral therapy (HAART) to minimise mortality, there will be escalating stress on health provision. Once HAART has transformed HIV from an acute to a chronic illness, patients must be supported in adhering to treatment so that they do not accumulate resistant virus which can once again impair immunity and result in acute illness from opportunistic infections. Further, because chronic HIV infection results in various forms of organ damage, and because PLHIV are as vulnerable to unconnected illnesses as other people, it is also essential to ensure their access to general health facilities. The main barrier is the attitude of health workers: they may be afraid of HIV infection, and may stigmatise patients known or thought to carry it. Irrational fears and discriminatory attitudes can be addressed through training, but health care workers need to be trained and equipped to prevent cross-infection between those with HIV and uninfected staff or patients, so as to alleviate unjustified fears and prevent nosocomial transmission. The paper also considers current strategies to limit transmission from PLHIV, including the use of legislation. Social ramifications of high HIV prevalence (for instance loss of key professional cadres, economic losses, or orphaning) are not covered because they result mainly from untreated infections rather than from diagnosed and stabilised chronic cases.

### Extending HAART coverage to an expanding caseload

When symptomatic HIV cases start to emerge in numbers, the total cost of managing and treating a national caseload quickly becomes substantial, because treatment involves lifelong intake of recently developed drugs and regular monitoring of their continuing efficacy.

An increasing proportion of the HIV caseload in developing countries is now able to access free-of-charge treatment. However, most governments of countries with generalised epidemics will find it challenging to cover the future costs of treating the expanding numbers of PLHIV, especially now that the recommended thresholds for commencing treatment have risen. At present most high prevalence countries can treat only a minority of those who meet the clinical criteria, even with substantial external assistance. Over the next few years, with donor economies providing less assistance due to the economic downturn[2], budgets will shrink as demand for HAART grows[3].

The availability of low-cost generic ARVs from India's pharmaceutical industry has been critically important to developing country treatment programmes over the last few years, rendering mass treatment achievable. According to the Office of the High Commissioner for Human Rights, 89% of 2010 supplies to donor-funded HAART programmes were Indian generics[4-6], alongside 80% of the ARVs used by Médecins Sans Frontières and the majority of ARVs supplied through the US government's PEPFAR programme[7]. India's 2005 accession to the World Trade Organisation (WTO) and resulting signature of the TRIPS (Trade-Related Aspects of Intellectual Property Rights) agreement commenced the alignment of national patent legislation with WTO standards. During the transition period, India has used the public health provisions of TRIPS, as agreed in the Doha Round, to maintain export of generic ARVs.

Now a more restrictive free trade agreement with the European Union is being negotiated, to increase protection for the international pharmaceutical industry through strengthening of intellectual property laws. The change would extend and enhance patent protection for branded drugs and thus shut down legal production of some ARVs in India. Fortunately the Indian government has successfully resisted the incorporation of the TRIPS data exclusivity clause, but discussions on other elements continue[8]. Total costs for universal treatment at national level are likely to increase substantially as the use of stavudine is discontinued[9] due to its toxicity[10,11], and as longer-term patients who have developed resistant virus need to be switched to second-line regimens. A recent study in South Africa[12] determined the cost of using tenofovir to be about five times that of the stavudine it replaces. Meanwhile, the manufacturers of the patented versions of many of the commonly used ARVs are currently reducing the level of discounting they offer to middle-income countries[13].

If the extra money cannot be found to pay higher prices for ARVs, and the lobbying efforts of the Access to Essential Medicines Campaign do not succeed, then formal commitments to full free-of-charge treatment coverage may have to be revised. Botswana has indicated that it has already reached this position, with more PLHIV expected to fund their own treatment in the future, to spare government funds for other pressing needs[14].

### Supporting adherence to preserve access to affordable drugs

A patient diagnosed with HIV infection must commence a daily regimen of pills when CD4 cell levels fall below a certain threshold. Many PLHIV would prefer to keep their condition confidential. Ensuring the required level of adherence (with full adherence defined as all treatments taken within an hour of the correct time, every day) is hampered by inability to take the pills when others might observe and guess why they are needed[15-17]. For the older regimens mainly used in the developing world, adherence poorer than 90-95% is likely to result in the development of resistant virus[18-23], while treatment interruptions can also encourage resistance[24].

Patients not fortunate enough to be admitted to a funded programme must find a way to pay for their treatment themselves. Costs can be substantial, with one study in Uganda finding that each clinic visit represented approximately 10% of the monthly wage for men, and 20% for women[25,26]. Studies in Botswana, Senegal, Cote d'Ivoire[27] and Uganda[28] have analysed the reasons behind low adherence in resource-poor populations where patients must pay towards the cost of their treatment, and in each of these cases the main reason stated by patients was the cost of purchasing their medication. Financial barriers may rise after a patient's condition is stabilised: when a patient has been critically ill relatives will contribute to transport costs, but this can rarely be sustained once the illness has become a chronic but not immediately life-threatening condition[25].

Patients who are unable to find the funds to renew their prescription will discontinue treatment temporarily or permanently, or somehow reduce the cost of their ARV regimen, for instance by purchasing one or two ARVs rather than the three needed to protect against resistance. Where monotherapy or duotherapy is undertaken in place of triple therapy as a cost-saving measure, resistance will develop much faster, as shown by an Indian study. India has now commenced free first-line treatment for 340,000 Indian PLHIV, but many patients have for some time self-funded treatment by private practitioners. In a study of 279 Mumbai patients purchasing ART, a fifth (54/279) were receiving mono-or duotherapy, prescribed by private practitioners to allow them to sell affordable ART for less than the cost of triple therapy[29]. These drugs are prohibitively expensive for many: patients who were able to buy treatment reported spending a median of 60% of their monthly income on their ARVs. Patients who achieved 95% adherence or better were three times more likely to register viral load below 400 copies/mL than those who did not, and patients on HAART were more than five times as likely to achieve this level of viral control compared to those taking mono-or duotherapy. More than a quarter (27%) had not managed to take at least 95% of their treatment on time, while 30% showed a rebounding viral load.

Treatment free of charge facilitates patient uptake of HAART, but failures in the supply line for antiretrovirals may also interrupt adherence[30]. In June 2011, Ghana had to draw down emergency supplies of ARVs priced at USD1.5 million[31], and in July 2011 protests occurred in Algeria[32] and Swaziland[33] over ARV supply problems. Supply interruptions result from seasonal or other transport breakdowns, inadequate systems, understaffing, and weak management of supply systems and personnel. Lack of funds available centrally for purchase of the drugs also causes supply interruptions, for instance because of delays in release of funds from donor or government budgets. One of the ways that patients react to these interruptions is to arrange to share the ARVs of others who are on treatment. This can result in both donors and recipients lacking enough drugs to maintain adequate adherence[29].

In poor countries especially, the acquisition of resistant strains may result in patients quickly exhausting the affordable options for treatment. Such patients might thus become effectively untreatable unless they can access the costlier newer ARVs to prevent them from relapsing from a controlled chronic infection into an immunodeficient state which results in the development of AIDS. It may also increase the possibility of them transmitting resistant infection to others through vertical (mother-to-child), sexual or medical routes.

### Demands on health services

In addition to the considerable infrastructure needed to deliver and monitor antiretroviral treatment, and to address the metabolic disturbances that it causes (some of which are life-threatening), a caseload of chronic HIV patients implies other challenges for health services. Treatment of the gradual non-infectious health damage done by HIV is needed, especially in relation to the cardiac[34], renal[35] and neurological[36] damage caused by persistent inflammatory responses. These morbidities are reduced by HAART in some but not all cases[35]. Referral to mainstream health care facilities for these and for unrelated medical problems will often be necessary; in many countries mainstream clinicians are afraid of treating people with HIV[37]. A Pakistani PLHIV reported

*"When I take people with AIDS to the hospital, doctors will wear two and sometimes three pairs of gloves (and) will stay as far away from them as possible. If doctors are so uncomfortable around us, what can you expect from those less knowledgeable?"*[38]

Protection of front-line health care staff from contracting dangerous infections at work is a prerequisite for compassionate care for their patients. However, where providers can limit their own perceived risks of contracting HIV to levels they find acceptable, they may still exclude PLHIV from fee-for-service facilities, due to community stigma. Their presence may deter other paying customers who will fear infection if they become aware that PLHIV are treated in the same facilities[39]. The staff may then find their institutional and personal income greatly reduced, and they may be reluctant to risk this situation by accepting PLHIV as patients[25]. Some healthcare workers hold inappropriate beliefs about the need for isolation of HIV-positive people to protect other patients[40].

It is important for health care staff to have the supplies needed to practice universal precautions. Poor infection control puts other patients at risk as well as practitioners: a recent study in Mozambique found that of HIV-positive children aged 0-11 years old, 31% of the mothers were seronegative[41], with a significant correlation between seropositivity and having received a medical injection in the last year[42]. In Swaziland, 3% of 1665 children aged 2-12 sampled in a general population were HIV-positive, and 22% of these had seronegative mothers[43]. Studies in Congo-Kinshasa[44] and South Africa[45] made similar findings.

Patients with chronic HIV who have low CD4 counts are also at risk from poor infection control. For those with very recent or well-controlled infection the risks may be slight, but if they have not been able to access ART or if their adherence has been inadequate to control viral load, they are at risk of acquiring opportunistic infections from unhygienic health facilities.

However, since the advent of "structural adjustment" cuts to health budgets, it is common for developing country health care systems to be short of of gloves and disposable equipment, and to lack the means for sterilisation of reusable equipment and disposal of contaminated sharps[46,47]. WHO has acknowledged that the sterilisation procedures for reused medical equipment are inadequate in many developing countries[48].

Medical staff may be able to spearhead the necessary change in attitudes to PLHIV, once they themselves have received appropriate training in infection control and stigma awareness. There is evidence that interacting with people who are living with HIV can reduce stigma among both health workers and the general public[49-51].

### Limiting prevalence of chronic HIV over the coming decades

Limiting the numbers of chronic HIV cases demands an active engagement with effective prevention, to reduce the numbers of new cases who will need ART in a few years. In 2009, according to UNAIDS, there were 2.6 million [2.3-2.8 million] new infections, representing an 8% annual increase in caseload[1].

Some countries have introduced HIV-specific criminal laws to try to reduce infection. While these are appropriate to prevent medical transmission, they pose many problems when applied to sexual and vertical transmission. Not only is there no evidence to prove that such laws do in fact reduce the spread of HIV, but the legislation is often ill-drafted and may for instance: accidentally criminalise conception (e.g. Guinea-Conakry, Guinea-Bissau, Mali, Niger, Kenya); breach medical confidentiality requirements by enabling or requiring those carrying out tests to disclose a patient's HIV+ status to known or suspected sexual partners (e.g. Benin, Kenya, Democratic Republic of Congo, Mali, Niger, Tanzania, Togo, Moldova and Papua New Guinea); or block sex education to young people, (e.g. Guinea-Conakry and Mali)[52].

Malicious transmission is already illegal in every jurisdiction of the world under provisions outlawing deliberately harming other people[53], so HIV-specific provisions covering malicious sexual transmission or exposure are redundant. It is inappropriate to place responsibility for blocking further spread of the epidemic on to people with chronic infection who have already been diagnosed and treated: HIV transmission cannot be blocked by controlling their behaviour or reducing their liberty because the bulk of transmission occurs from those not yet on treatment, especially those in primary infection (accounting for 46.5% of all new infections in the Ugandan Rakai study[54]). Not only is it doubtful whether HIV can be passed on from PLHIV whose antiretroviral therapy has succeeded in reducing their blood viral load to undetectable levels[55], research shows that one of the effects of HIV diagnosis on PLHIV is a focus on trying to reduce the possibility that the infection could be passed on[34,56]. Thus for effective prevention, attention should be concentrated on people not so far diagnosed as PLHIV, who behave in ways that are likely to cause them to contract HIV and to pass it on once they are infected.

Behaviour change intervention at a population level is needed. Lessons must be learned about what works: for instance models that rely on the individual-focused health belief model have not proved particularly successful because of inattention to barriers to uptake and to the impact of local social norms. The widely used ABC model (abstain, be faithful, use condoms) has had mixed results, tending to poor results where the C was omitted from health promotion efforts. The focus of design of prevention programmes should be on understanding how traditional and modern belief structures impact upon behaviour that poses risks to self or to others, in order to modify prevention messages so that they generate behaviour change and not stigma. Allen et al present a more nuanced model for secondary prevention, based on operational experience, developed in Uganda by TASO[57]; this study highlights some of the challenges involved.

Incorrect and irrational beliefs about the causation of AIDS thrive across the developing world, and can block assimilation of evidence-based health messages on HIV even where these are accurately communicated to the population. More sophisticated approaches are needed, focused on communicating insight into personal risks and on modifying social norms. All HIV programming should incorporate stigma reduction checks at every level, as fear of the consequences of exposure of seropositivity is among the most common reasons for loss of prevention programme impact as well as treatment take-up and success[29].

## Conclusions

In higher prevalence countries especially, the demands of managing the response to HIV are heavy, requiring coordination between ministries of health, donors, logistics teams and local service delivery points. Supportive laws, non-discriminatory health care provision, robust infection control and reliable drug supply chains are all needed to support care and treatment for the caseload of chronic HIV patients. Attention to the affordability of ARVs is crucial. The decision of the Botswana government to leave many patients with chronic HIV to fund their own treatment through the private sector may prove to be expensive in cost, morbidity and even mortality in the long term if higher drug resistance results, as seen in Mumbai. If patient contributions are essential, then it would be best for supply and adherence to be controlled centrally and contributions made through co-pays, rather than taking the easier option of leaving provision for these people to the private sector.

There is need to manage issues arising around secondary transmission and to develop HIV prevention and awareness programmes for the general public that result in better self-protection and less persecution of people known to have HIV. All health care workers need to be supported with information and supplies so that they can protect themselves and their patients from HIV transmission, work without fear, and provide a full and non-discriminatory service to those living with HIV as a chronic condition.

## Abbreviations

ART: Antiretroviral therapy; ARV: Antiretroviral medication; HAART: Highly active antiretroviral therapy (triple therapy); PLHIV: People living with HIV; WTO: World Trade Organisation.

## Competing interests

The author declares no competing interests. No funding was received for the preparation of this paper.
